# Estimating the nutrient thresholds of a typical tributary in the Liao River basin, Northeast China

**DOI:** 10.1038/s41598-018-22128-9

**Published:** 2018-02-28

**Authors:** Jiabo Chen, Fayun Li, Yanjie Wang, Yun Kong

**Affiliations:** 1 National & Local United Engineering Laboratory of Petroleum Chemical Process Operation Optimization and Energy Conservation Technology, Liaoning Shihua University, Fushun, 113001 China; 2 Institute of Eco-environmental Sciences, Liaoning Shihua University, Fushun, 113001 China; 3 Yixing Urban Supervision & Inspection Administration of Product Quality, China National Supervision & Inspection Center of Environmental Protection Equipment Quality (Jiangsu, preparation), Yixing, 214205 China

## Abstract

Estimating regional nutrient criteria for streams and rivers is a key step toward protecting river water quality and restoring the health of aquatic ecosystems. Using a multivariable statistical analysis technique, nutrients were identified as the main factor influencing the degradation of the benthic macroinvertebrate community. Three chemical methods (the reference stream distribution approach, all-streams distribution approach and Y-intercept approach) and one biological method (the stress-response approach) were applied to evaluate the nutrient thresholds in the Qing River basin. The reference stream distribution approach and all-streams distribution approach were based on calculating a predetermined percentile of reference streams and all-streams water quality data set, respectively. The Y-intercept approach was based on determining the influence of human activity on water quality by linear regression models. The biological method was based on the response of the benthic macroinvertebrate community structure to changes in water quality. The chemical thresholds were 0.750–1.288 mg/L for total nitrogen (TN) and 0.035–0.046 mg/L for total phosphorus (TP); the biological thresholds were 1.050–1.655 for TN and 0.052–0.101 for TP. The results from the chemical approaches were verified using the biological method, resulting in preliminarily recommended thresholds of 1.000 mg/L TN and 0.040 mg/L TP in the Qing River system.

## Introduction

Eutrophication is a common environmental problem throughout the world, primarily occurring in enclosed or semi-enclosed water bodies such as lakes, reservoirs and bays^1–4^. With the excessive exploitation of natural resources and the rapid development of industry and agriculture, the nutrient concentrations in rivers have increased in recent years^4–7^. Excessive nutrient levels can negatively impact river water and cause various problems, including affecting the health of humans and livestock, changing the structures of river ecosystems, and reducing the aesthetic and recreational features of the rivers^2,8–10^. Furthermore, because rivers are the source of water for many lakes, coastal waters and wetlands, increased nutrients may increase eutrophication in these downstream waters as well^4,8,11,12^.

Establishment of water quality criteria and standards is a key step toward protecting water quality^8^. A number of developed countries have been or are formulating nutrient criteria for surface waters. The United States Environmental Protection Agency (US EPA) issued a national strategy for developing a regional set of nutrient criteria in 1998^9^, and a nutrient criteria technical guidance manual for rivers and streams was published two years later^8^. Dodds *et al*.^3^ estimated the boundaries of oligotrophic, mesotrophic and eutrophic states of rivers using published data on the water quality of large- and medium-scale rivers in the United States with a frequency distribution method in 1998. In 2000, the US EPA^8^ published methods for evaluating reference conditions, including a reference stream distribution approach based on a predetermined percentile of the water quality data set of reference streams and an all-streams distribution approach based on a predetermined percentile of the water quality data set of the general stream population. Smith *et al*.^13^ evaluated the natural background concentrations of nutrients in streams and rivers of the contiguous United States by integrating nutrient yield models with the SPARROW transport model from existing reference streams. Dodds and Oakes^14^ employed a Y-intercept approach for establishing reference conditions across watersheds based on estimating the influence of land use on river nutrient concentrations by means of multiple linear regression analysis. Some researchers^15–17^ have evaluated the environmental thresholds for nutrients in streams using a stress-response approach based on the relationships between biological response parameters and river water quality parameters.

The research on water quality criteria and standards is sparse in China compared with developed countries. Water quality criteria are mainly derived from the relevant works in foreign countries^18^, which reduces the applicability of these criteria due to the regional variability of aquatic biota^19^. Nutrient levels and reactions vary greatly across a country, and the US EPA has suggested that recommended nutrient criteria must reflect the characteristics of different geographical regions and types of water bodies^9^.

The existing Chinese surface water environmental quality standards (GB3838-2002) have wide coverage and poor regional specificity, which may result in problems of under- or over-protection for river water management. In addition, controls on total nutrient quantities consider mainly point source parameters in China; few consider non-point source parameters. The water quality standards lack total nitrogen (TN) control standards for streams and rivers, and as such, they cannot address the main causes of nutrient pollution in water bodies via non-point source pollution. Establishing regional nutrient criteria and standards is a fundamental step toward reducing nutrient pollution in aquatic ecosystems in China.

The study of nutrient criteria for streams and rivers is still in its infancy in China. Wu *et al*.^20^ (2010) have proposed Chinese water environment quality criteria. Chen and Lu^21^ have established nutrient criteria for streams and rivers with three chemical methods in the Cao-E River basin. Wu *et al*.^22^ have estimated river nutrient thresholds based on benthic macroinvertebrate assemblages in the upper reaches of Xitiao Stream in Zhejiang, China. However, there are few comparative studies of nutrient criteria for streams and rivers based on chemical and biological approaches in China.

Exploring the influence of river basin characteristics on river water quality can help identify the main pollutants that affect benthic macroinvertebrate assemblages and evaluate the nutrient thresholds. According to the geographical features of Northeast China, nutrient criteria of streams and rivers were evaluated in the Qing River system using a combination of chemically derived threshold approaches (the reference stream distribution approach, all-streams distribution approach, Y-intercept approach) and a biologically derived threshold approach (stress-response approach). Each of these approaches has its own application scope and shortcoming^16,21^. The chemically derived thresholds were cross-calibrated with the biologically derived thresholds. The use of these three chemical methods and one biological method exploits the advantages and avoids the disadvantages of each method. These four complementary methods can provide strong support for the establishment of water quality criteria and standards in China and scientific and technological support for the implementation of water pollution controls.

## Results and Discussion

### Influence of river basin characteristics on river water quality

**Figure 1:**
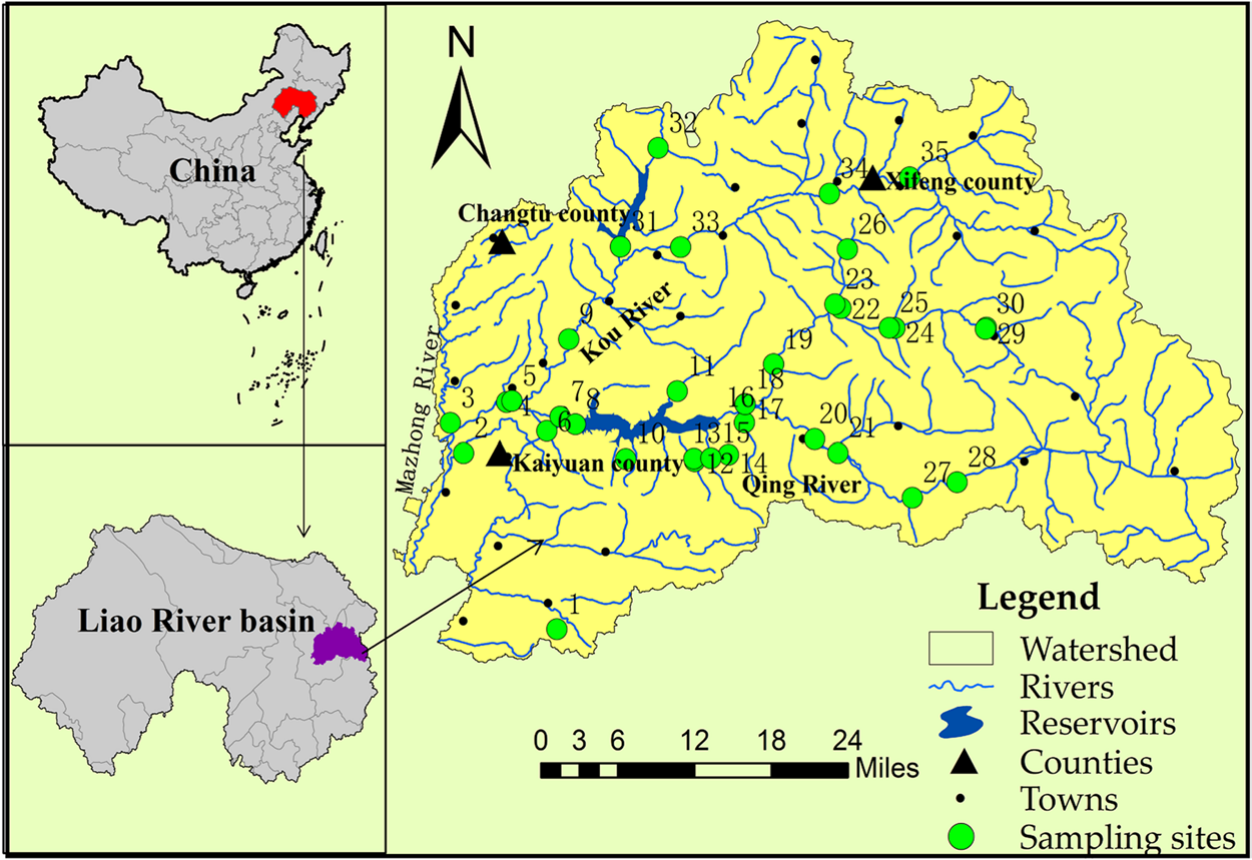
Locations of sampling stations. Latitude and longitude were measured using a hand-held GPS unit, and ArcGIS 10.0 Desktop GIS software (http://www.esri.com/arcgis/about-arcgis) was used to plot the sampling stations.

The river basin characteristics, the river water quality parameters and the potential relationships between them at the sampling sites are discussed to identify the main pollutants and further assist in evaluating the nutrient thresholds in the Qing River basin. Table 1 gives the descriptive statistics for the physical and chemical parameters of the water, the river habitat assessment indicators and land use. Sulfate and pH exhibited less spatial variability than the other physical and chemical parameters, with coefficients of variation of 22.27% and 8.06%, respectively. The fecal coliform count (FCC), total bacterial count (TBC) and total phosphorus (TP) exhibited more spatial variability, with coefficients of variation of 134.08%, 100.21% and 100.00%, respectively. Sites with higher TP concentrations were located in the middle and lower reaches of the Qing River (monitoring sites 3, 13, 16 and 34; Fig. 1).

**Table 1:** Characteristics of the Qing River.

Parameter	Mean	SD	CV	Range	Parameter	Mean	SD	CV	Range
**Habitat Descriptor**					**Physical-Chemical Parameter**				
Epifaunal Substrate⁄Available Cover	13	4	30.77	5–18	TP (mg/L)	0.07	0.07	100.00	0.01–0.33
Embeddedness	14	5	35.71	4–19	TN (mg/L)	1.16	0.74	63.79	0.15–3.64
Velocity/Depth Regime	13	4	30.77	5–18	NH_4_^+^-N (mg/L)	0.50	0.36	72.00	0.07–1.62
Sediment Deposition	14	4	28.57	6–18	NO_3_^−^N	1.346	0.747	55.50	0.55–2.21
Channel Flow Status	13	4	30.77	6–18	COD_Cr_ (mg/L)	16.2	7.8	48.15	4.5–42.0
Channel Alteration	12	5	41.67	4–19	BOD_5_ (mg/L)	3.2	1.1	34.36	2.0–7.9
Frequency of Riffles	13	5	38.46	4–18	DO (mg/L)	8.5	2.2	25.88	5.5–15.6
Bank Stability	13	4	30.77	7–18	pH	7.82	0.63	8.06	6.26–9.26
Vegetative Protection	12	4	33.33	4–18	EC (μS/cm)	330	143	43.33	128–714
Riparian Vegetative Zone Width	12	5	41.67	4–19	T (°C)	24.6	3.6	14.63	17.6–30.9
Total Rapid Bioassessment Protocol (RBP) Score	131	44	33.59	49–181	Chloride (mg/L)	22.47	13.4	59.64	10.9–68.9
**Land Use**					Sulfate (mg/L)	48.5	10.8	22.27	33.4–75.7
Cropland (%)	26.6	22.7	85.34	5.0–80.0	FCC (num/L)	723.4	969.95	134.08	90–2300
Urban Land (%)	2.0	2.7	135	0.2–15.0	TBC (num/mL)	1397	1400	100.21	150–5500
Forest Land (%)	70.7	24.7	34.94	9.0–93.6	Molar N:P	58	51	87.93	7–267
Population Density	112	146	130.36	20–800

The average values of the NH_4_^+^-N concentration, COD_Cr_, BOD_5_ and FCC (Table 1) were 0.5 mg/L, 16.2 mg/L, 3.2 mg/L and 723.4 num/L, representing grade II, grade III, grade III and grade II, respectively, based on the environmental quality standards for surface water in China (GB3838-2002). The sub-watersheds of the 35 monitoring sites in the Qing River system had high land use variability. The ion concentration, represented by the EC, was very significantly associated with the chloride concentration, sulfate concentration and land use area percentage and significantly associated with the TP concentration (Table 2). Though the EC variations were mainly determined by geographical differences, increased EC values are usually an indicator of urbanization^23,24^, which was confirmed by the strong relationship between the chloride concentration and EC in the Qing River basin. The sampling sites with higher nutrient concentrations usually had higher EC values because poor runoff management led to higher nutrient and ion concentrations. It has been shown previously that EC usually increases with the urban land area percentage, even in limestone regions with high background EC levels^17^.

**Table 2:** Pearson correlation coefficients among the water chemistry parameters, habitat parameters and land uses at the sampling stations in the Qing River basin.

Parameter	TP	TN	NH_4_^+^-N	COD_Cr_	BOD_5_	DO	pH	EC	NO_3_^−^N	Chloride	Sulfate	FCC	TBC
**Physical-Chemical Parameter**													
TP	1												
TN	0.226	1											
NH_4_^+^-N	0.295	−0.077	1										
COD_Cr_	0.711**	0.409*	0.474**	1									
BOD_5_	0.707**	0.151	0.187	0.680**	1								
DO	0.164	−0.433*	0.198	0.228	0.355*	1							
pH	0.027	−0.5	0.306	0.006	−0.014	0.711**	1						
EC	0.420*	0.190	0.785**	0.558**	0.377**	0.080	−0.008	1					
NO_3_^−^N	−0.312	0.646**	0.433	0.404	0.068	−0.055	−0.146	−0.561*	1				
Chloride	0.250	0.461*	0.908**	0.562*	−0.101	−0.575**	−0.323	0.894**	0.544*	1			
Sulfate	0.221	0.690**	0.755**	0.708**	−0.128	−0.534*	−0.682**	0.829**	0.447	0.736**	1		
FCC	0.212	0.047	0.183	0.149	0.332	−0.343	−0.367	0.396	0.375	0.393	0.305	1	
TBC	−0.027	0.608**	0.377	0.137	−0.063	−0.231	−0.202	0.406	0.314	0.256	0.449	−0.004	1
**Habitat Descriptors**													
Epifaunal Substrate⁄Available Cover	−0.35*	−0.226	−0.313	−0.539**	−0.302	−0.105	−0.029	−0.293	−0.257	−0.659**	−0.711^**^	−0.100	−0.463*
Embeddedness	−0.378*	−0.372*	−0.377*	−0.574**	−0.326	−0.093	−0.024	−0.372*	−0.253	−0.659**	−0.697^**^	−0.060	−0.493^*^
Velocity/Depth Regime	−0.354*	−0.329	−0.266	−0.507**	−0.332	−0.106	−0.020	−0.267	−0.235	−0.609**	−0.673^**^	−0.088	−0.382
Sediment Deposition	−0.374*	−0.376*	−0.341*	−0.571**	−0.356*	−0.117	−0.035	−0.318	−0.258	−0.645**	−0.669^**^	−0.116	−0.508^*^
Channel Flow Status	−0.354*	−0.366*	−0.304	−0.537**	−0.338*	−0.092	−0.006	−0.317	−0.245	−0.578**	−0.683^**^	−0.106	−0.431
Channel Alteration	−0.416*	−0.381*	−0.275	−0.517**	−0.362*	−0.048	0.016	−0.303	−0.225	−0.573*	−0.676^**^	−0.045	−0.392
Frequency of Riffles	−0.352*	−0.345*	−0.252	−0.518**	−0.338*	−0.111	−0.010	−0.259	−0.263	−0.595**	−0.708^**^	−0.086	−0.443
Bank Stability	−0.346*	−0.343*	−0.216	−0.482**	−0.309	−0.092	−0.014	−0.227	−0.196	−0.511*	−0.674^**^	−0.076	−0.369
Vegetative Protection	−0.366*	−0.354*	−0.293	−0.533**	−0.333	−0.128	−0.053	−0.293	−0.229	−0.602^**^	−0.690^**^	−0.066	−0.417
Riparian Vegetative Zone Width	−0.354*	−0.369*	−0.293	−0.519**	−0.311	−0.102	−0.033	−0.295	−0.187	−0.573^*^	−0.686^**^	−0.033	−0.448
**Land Use**													
Cropland	0.497**	0.650**	0.374*	0.664**	0.435**	−0.080	−0.196	0.524**	0.308	0.717**	0.698**	0.088	0.632*
Urban Land	0.779**	0.105	0.527**	0.738**	0.751**	0.368*	0.121	0.623**	0.322	0.820**	0.356	0.313	0.234
Forest Land	−0.549**	−0.615**	−0.408*	−0.698**	−0.484**	0.038	0.169	−0.56**	−0.312	−0.738**	−0.693**	−0.100	−0.623**
Population	0.813**	0.074	0.520**	0.788**	0.832**	0.388*	0.153	0.571**	0.118	0.536*	0.166	0.309	0.099

^*^Indicates significance at the 0.05 probability level.

^**^Indicates significance at the 0.01 probability level.

The average river habitat score of the sub-watersheds was 131 (total score of 200); the lowest average scores (12) were for channel alteration, vegetative protection and riparian vegetative zone width (Table 1), which showed that these parameters were the most limiting river habitat parameters in the Qing River basin. The highest average scores (14) were for embeddedness and sediment deposition, showing that these parameters were at acceptable levels. The river habitat parameters were very significantly negatively associated with COD_Cr_ and significantly negatively associated with the nutrient concentrations (TN or/and TP), and the TN concentration was very significantly associated with the NO_3_^−^N concentration (Table 2), which indicated that the main pollution type was non-point source. The TN concentration was higher than the TP concentration by multiple orders of magnitude, and the molar N:P ratios at most of the sampling sites were greater than 17 (Table 1).

The TN concentration, EC, COD_Cr_ and BOD_5_ were very significantly associated with the main land use percentage in the Qing River system (Table 2), which indicated that land use made the greatest contribution to the nutrient, ion, and oxygen consumption in the River. The total habitat scores decreased as the urban land and cropland percentages increased (Table 2). The nutrient concentrations in the river water increased with increasing human activities. The correlation analysis (Table 2) showed that the TP level was closely associated with the urban land, cropland and woodland percentages (r values of 0.779, 0.497 and −0.549, respectively). Higher ion and TP concentrations (monitoring sites 3, 13, 16 and 34; Fig. 1) were found near urban land (wastewater treatment plant outflow, domestic wastewater and livestock wastewater). The TN concentration was very significantly associated with the cropland and woodland percentages (r values of 0.650 and −0.615, respectively) because the research area is mainly affected by agricultural non-point source pollution^21,24–27^. Poor agricultural management practices and mining activities can input suspended solids and sediments, including high levels of nutrients into rivers^21,25,28^. pH had a low correlation with the urban land percentage (r value of 0.121) mainly because the area was located in the agricultural region of the Qing River basin (the research area contained little urban land, and most of the urban land was located downstream of the Qing River basin). The relationship between land use and nutrient concentrations was explored by multiple regression analysis, and the results (Table 3) showed that cropland and population density could explain 42.3% and 66.1% of the variance of TN and TP, respectively. The cropland and residential domestic pollution contributed substantially to the increased river nutrient concentrations.

**Table 3:** Results of the regression analyses between nutrient (TN and TP) concentrations and land uses in the Qing River basin.

Model Parameter		TN					TP		
		bi	Standard Error	t	P value	bi	Standard Error	t	P value
Independent Variable	Population Density (people km^−2^)	~	~	~	~	0.334	0.042	8.028	0.000
	Urban Land Area (%)	~	~	~	~	~	~	~	~
	Cropland Land Area (%)	1.891	0.385	4.918	0.000	~	~	~	~
Intercept (b_0_)		0.749	0.134	5.604	0.000	34.717	7.578	4.581	0.000
Model R^2^		0.423				0.661			

### Relationship between benthic macroinvertebrate community characteristics and river nutrient concentrations

Benthic macroinvertebrates, the most commonly used environmental indicator organisms in biological water quality monitoring, can represent the cumulative effect of water body disturbance and pollution stress in a past period of time; many studies^16,17,22^ indicated that nutrient pollution can negatively affects the benthic macroinvertebrate community structure. A total of 70 benthic macroinvertebrate taxa belonging to 11 orders (Diptera, Ephemeroptera, Trichoptera, Plecoptera, Coleoptera, Hemiptera, Decapoda, Basommatophora, Gnathobdellida, Rhynchobdellida, Oligochaeta: Plesiopora) and three phyla (Annelida, Mollusca, Arthropoda) were collected over the study period. The relative abundance of taxonomic groups at the order level revealed that the most abundant taxa in the study area, especially downstream, were in the order Diptera (37.52%). Ephemeroptera (26.16%) and Trichoptera (19.23%) were the next most abundant orders in the study area and were the most represented groups in the forest areas. Four biological metrics, which are based on the evaluation of macroinvertebrate measures and are commonly used for assessing water quality and river ecosystem health, were significantly related to the TN and TP concentrations (Table 4) and were calculated for all the sampling stations (Fig. 1): the Ephemeroptera, Plecoptera, and Trichoptera (EPT) relative abundance, EPT taxonomic richness, modified family biotic index (FBI), and Diptera and non-insect relative abundance. The EPT relative abundance percentages (range of 8–74%) and EPT taxonomic richness (range of 1–18) were higher at monitoring sites 27–30 (Fig. 1). The modified FBI values (range of 3.79–6.79) were the highest at monitoring sites 2–5. The Diptera and non-insect relative abundance percentages ranged from 16% to 86%, were the lowest at monitoring sites 27–30 and 32 (16–19%) and the highest at monitoring sites 2–5 (76–86%).

**Table 4:** Pearson correlation coefficients between the environmental variables and benthic macroinvertebrate metrics.

Parameter	EPT Relative Abundance (%)	EPT Taxonomic Richness	Modified FBI	Diptera and Non-Insect Relative Abundance
**Physical-Chemical Parameter**				
TP	−0.430^**^	−0.433^**^	0.383^*^	0.406^*^
TN	−0.409^*^	−0.398^*^	0.404^*^	0.424^*^
NH_4_^+^-N	−0.374^*^	−0.363^*^	0.406^*^	0.384^*^
BOD_5_	−0.365^*^	−0.321	0.291	0.375^*^
DO	−0.056	−0.021	0.029	0.050
pH	0.010	0.023	−0.002	−0.026
EC	−0.368^*^	−0.410^*^	0.388^*^	0.399^*^
T (°C)	0.153	0.152	−0.156	−0.162
NO_3_^−^N	−0.363	−0.280	0.302	0.387
FCC	−0.147	−0.187	0.134	0.144
TBC	−0.459^*^	−0.383	0.434	0.449
**Land Use**				
Cropland	−0.459^*^	−0.704^**^	0.741^**^	0.799^**^
Urban Land	−0.459^*^	−0.506^**^	0.502^**^	0.551^**^
Forest Land	0.771^**^	0.710^**^	−0.743^**^	−0.801^**^
Population	−0.494^**^	−0.455^**^	0.453^**^	0.502^**^

**Figure 2:**
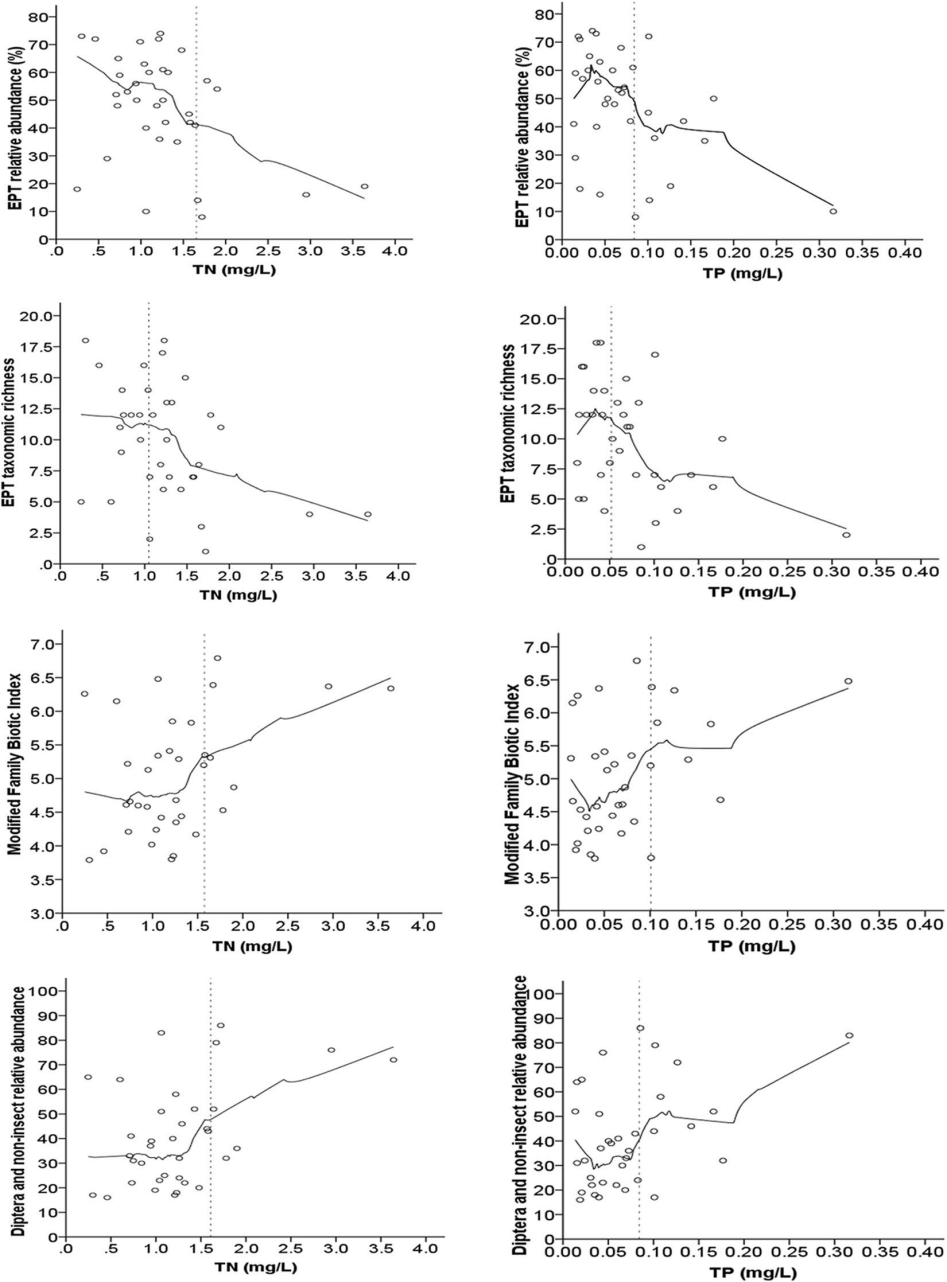
Relationships between the benthic macroinvertebrate metrics and nutrient (TN and TP) concentrations in the Qing River basin. The smooth lines show the locally weighted scatterplot smoothing (LOWESS).

Unbalanced levels of nutrients, ions and DO are the possible causes of biological degradation in rivers^29,30^. The results (Table 4) showed that DO was not significantly related with the 4 benthic macroinvertebrate indices. Although the indices were significantly associated with EC, these correlations were weaker than their correlations with the nutrient concentrations (TN and TP). TP and TN were significantly associated with the EPT relative abundance % (r = −0.430, p < 0.01, and r = −0.409, p < 0.05, respectively), EPT taxonomic richness (r = −0.433, p < 0.01, and r = −0.398, p < 0.05, respectively), modified FBI (r = 0.383, p < 0.05, and r = 0.404, p < 0.05, respectively) and Diptera and non-insect relative abundance (r = 0.406, p < 0.05, and r = 0.424, p < 0.05, respectively), and TP also had very significant relationships with the EPT relative abundance % and EPT taxonomic richness (Table 4). The EPT relative abundance % (r = −0.459, p < 0.05, and r = 0.771, p < 0.01, respectively), EPT taxonomic richness (r = −0.704, p < 0.01, and r = 0.710, p < 0.01, respectively), modified FBI (r = 0.741, p < 0.01, and r = −0.743, p < 0.01, respectively) and Diptera and non-insect relative abundance (r = 0.799, p < 0.01, and r = −0.801, p < 0.01, respectively) had significant relationships with the percentages of cropland and woodland (Table 4), which showed that the substantial nutrient loss caused by agricultural cultivation has altered the community structure of benthic macroinvertebrates. As shown in Fig. 2, the biotic indices varied considerably at low nutrient concentrations but varied only slightly at high concentrations. The EPT relative abundance % and EPT taxonomic richness increased first and then decreased as the TP concentration increased. The EPT relative abundance % and EPT taxonomic richness decreased as the TN concentration increased. The modified FBI and Diptera and non-insect relative abundance increased as the TP and TN concentrations increased. Nutrients were not the main factors affecting the aquatic species distributions at low concentrations, and at high concentrations, they obviously hindered the growth of certain species; these results are similar to those of previous studies^16,22^.

### Nutrient criteria evaluation

Based on the Y-intercept approach^14^, the TP criterion was 0.035 mg/L with a 95% confidence interval of 0.019–0.050 mg/L in the Qing River system, and the TN criterion was 0.749 mg/L with a 95% confidence interval of 0.477–1.021 mg/L (Table 5). The TN and TP thresholds evaluated according to the reference stream distribution approach were 1.288 mg/L and 0.046 mg/L, respectively; the TN and TP thresholds evaluated using the all-streams distribution approach were 0.724 mg/L and 0.024 mg/L, respectively (Table 6). Nutrient criteria can be evaluated with a stress-response approach based on benthic macroinvertebrate community characteristics. Because the EPT relative abundance %, EPT taxonomic richness, modified FBI and Diptera and non-insect relative abundance play important roles in river health assessments^16,31,32^, these four biotic indices that were significantly correlated with both TN and TP were selected to establish the river nutrient criteria. The number of pollution-sensitive species decreased rapidly, and the stability of the community structure deteriorated when the nutrient (TN and TP) concentrations exceeded their respective thresholds (Fig. 2). Regression tree analysis with the four biotic indices (EPT relative abundance %, EPT taxonomic richness, modified FBI and Diptera and non-insect relative abundance) showed that the evaluated TN thresholds were 1.655 mg/L, 1.050 mg/L, 1.574 mg/L and 1.610 mg/L, respectively, and the evaluated TP thresholds were 0.084 mg/L, 0.052 mg/L, 0.101 mg/L and 0.084 mg/L, respectively (Fig. 2, Table 6). Among the evaluated nutrient thresholds obtained using the four approaches, the values determined with the stress-response approach were highest, followed by those determined with the reference stream distribution approach, the Y-intercept approach and the all-streams distribution approach, in decreasing order (Table 6).

**Table 5:** Predicted nutrient (TN and TP) thresholds and their confidence intervals determined using the Y-intercept approach for the Qing River basin.

Parameter	Threshold (mg/L)	n	Low 95% Confidence Interval (mg/L)	High 95% Confidence Interval (mg/L)
TN	0.749	35	0.477	1.021
TP	0.035	35	0.019	0.050

**Table 6:** Evaluated nutrient (TN and TP) thresholds for the Qing River basin in Northeast China.

Nutrient Threshold Evaluation Approach	Nutrient Threshold	
	TN (mg/L)	TP (mg/L)
**Chemically Derived Approaches**		
Reference Stream Distribution Approach	1.288	0.046
All-Streams Distribution Approach	0.724	0.024
Y-Intercept Approach	0.749	0.035
**Preliminarily Recommended Thresholds**	1.000	0.040
**Biologically Derived Approach**		
Stress-Response Approach based on Benthic Macroinvertebrate Community Indices		
EPT Relative Abundance %	1.655	0.084
EPT Taxonomic Richness	1.050	0.052
Modified FBI	1.574	0.101
Diptera and Non-Insect Relative Abundance	1.610	0.084

There are few reference streams or reaches in the developed region, and the nutrient loads of reference streams or reaches can vary significantly in the same ecological region due to differences in runoff^13,14,33^. The 5–25% frequency distribution for all streams was selected to evaluate the nutrient thresholds^9^. This 5–25% frequency distribution was a deduced value, and the selected frequency distribution can generate nutrient thresholds that result in under- or over-protection of the water quality^34^. The Y-intercept approach is simple and widely applicable and is primarily used in studies considering several major influencing factors, including land use and population density. However, river water quality is affected by many factors, such as livestock and poultry breeding^35–37^, point source pollution^24^, agricultural management practices^38,39^, atmospheric deposition^39,40^, topography^41,42^, and the riparian buffer^26,43,44^. In addition, the agricultural watershed is a region of internal change affected by human activities; even all human activities, including agricultural activities, were ceased, it is unclear whether nutrient outputs would be restored to their natural levels^16^. Some studies^16,45^ have indicated that the other factors might be equally or more important than nutrient levels in affecting the aquatic community structure of a certain area. In contrast, the stress-response approach based on biological attributes allows identification of nutrient thresholds consistent with good ecological condition; however, such models often have low explanatory power, especially for streams^14,16,45^.

Each nutrient criteria evaluation approach has disadvantages, and the comparison of the chemically derived thresholds (reference stream distribution approach, all-streams distribution approach and Y-intercept approach) and the biologically derived threshold (the stress-response approach based on benthic macroinvertebrate community structure) strengthens the validity of the nutrient criteria for the protection and rehabilitation of aquatic life. The nutrient thresholds estimated using the chemically derived approaches were 0.724–1.288 mg/L for TN and 0.024–0.046 mg/L for TP (Table 6). The chemically derived TN and TP thresholds could be used as the nutrient thresholds for the Qing River basin because they mainly lie within the bounds of all biological thresholds^16,17,32^. In addition, because the pollution in the study area can be categorized as have an agricultural non-point source at most sampling sites, the recommended preliminary nutrient thresholds are 1.000 mg/L for TN and 0.040 mg/L for TP (approximately equal to the averages of the chemical thresholds and less than the biological thresholds). Further work is required to evaluate other biological responses (e.g., algal and fish biological metrics) to nutrients and perform field evaluations of the applicability of the preliminary recommended nutrient thresholds.

## Materials and Methods

### Study area

The Qing River, a main tributary of the Liao River, is located in Northeast China. The Qing River basin is between N42.30°–42.70° and E123.80°–124.8° and has a catchment area of 4785 km^2^ (Fig. 1). The Qing River has a length of 217 km and 8 tributaries, including the Kou and Mazhong Rivers. The Qing River basin, with an average annual precipitation and temperature of 692 mm and 6.5 °C, respectively, has a temperate monsoon climate, with precipitation mainly occurring in the summer (July and August). The terrain is quite varied, from hills and mountains to alluvial plains. The vegetation coverage decreases from east to west. Land uses mainly include woodland, cropland, water and urban land. The eastern region of the Qing River basin, with little human activity, is predominantly woodland, and the rivers in this area have higher gradients, faster flows and a larger average grain size of riverbed sediments than those in the western region. Residential density, cropland, urban land percentage and human activities are all higher in the western region of the Qing River basin^46^. The riparian zone is more consolidated, and the river pollution load is higher in the urban areas. The Qing River basin includes Kaiyuan City, the Qinghe District and Xifeng County, with a population of over one million and an annual GDP of more than 20 billion Chinese yuan; the agricultural activities are dominated by cropland cultivation and animal husbandry in the Qinghe District^47^.

### Sample collection and river habitat investigation

The riparian characteristics, physical habitat parameters, hydrochemistry and biological integrity indices were evaluated in different periods in 2009 and 2011. A habitat survey was conducted utilizing the evaluation indices and methods for river habitats^48,49^; 10 indices were rated on a scale of 0–20 at the sampling sites, with high scores indicating a high-quality habitat. The monitored parameters included the TN, TP, ammonia nitrogen (NH_4_^+^-N), dissolved oxygen (DO), chloride, and sulfate concentrations; 5-day biochemical oxygen demand (BOD_5_); potassium bichromate index (COD_Cr_); pH; temperature (T); electrical conductivity (EC); FCC; and TBC. The sampling, preservation and analytical procedures were performed according to the national standard methods of China^50^; the analytical methods are listed in Table 7.

**Table 7:** Analytical methods used to assess water quality parameters of the Qing River basin.

Parameter	Analytical Method
TN	Alkaline potassium persulfate digestion with UV spectrophotometric detection
TP	Ammonium molybdate spectrophotometry
NH_4_^+^-N	Sodium reagent colorimetry
NO_3_^−^N	UV spectrophotometry
BOD_5_	Dilution and seeding
COD_Cr_	Dichromate method
DO	Portable DO analyzer
pH	pH meter
T	Portable water quality analyzer
EC	Portable water quality analyzer
Chloride	Ion chromatography
Sulfate	Ion chromatography
FCC	Multiple-tube fermentation
TBC	Dilution-culture method

Macroinvertebrate samples were collected at four locations within each of the 35 sites using a surber sampler with a size of 0.3 × 0.4 m and a pore diameter of 425 µm. The net was placed on rocky river substrates, and an area of 0.12 m^2^ upstream of the net was disturbed to dislodge and wash the macroinvertebrates into the net. Individual rocks in the sampled area were picked up, and the attached organisms were removed. The four replicate samples were combined, and all of the obtained aquatic organisms were preserved in 70% ethanol^51,52^.

### Derived nutrient thresholds

#### Reference stream distribution approach

The 75% frequency distribution of the nutrient data obtained for reference streams was applied to estimate the thresholds^8^.

#### All-streams distribution approach

The 5th to 25th percentile of the frequency distribution of the nutrient data obtained for all streams was selected to estimate the nutrient thresholds in the study area^8^.

#### Y-intercept approach

Multiple linear regression analysis was used to evaluate the thresholds. The logarithms of the in-stream nutrient concentrations are the dependent parameters, the land use percentages are the independent parameters, and the intercept of the regression model represents the nutrient thresholds^14^.

#### Stress-response approach

Based on the macroinvertebrate data, nine macroinvertebrate community indices were calculated: species richness, macroinvertebrate density, Shannon’s diversity index, the modified Shannon-Wiener index, evenness, EPT taxonomic richness, EPT relative abundance, the modified FBI, and the Diptera and non-insect relative abundance. Four community indices (EPT relative abundance %, EPT taxonomic richness, modified FBI and Diptera and non-insect relative abundance) were significantly correlated (p < 0.05) with both TN and TP and were therefore retained for further evaluation. EPT taxonomic richness is determined from the total number of taxa in the orders EPT^53^. EPT relative abundance is calculated as the sum of the number of taxa in the orders EPT divided by the total number of organisms in the sample^54^. The modified FBI considers organisms other than arthropods using genus- and species-level tolerance values and is calculated as follows in Equation (1)^55^:

1 $${\rm{FBI}}=\frac{{\sum }^{}{n}_{i}{t}_{i}}{N}$$

where *n*_*i*_ = individual number within a taxon, *t*_*i*_ = stressor tolerance of a taxon, and *N*=total number of organisms in the sample. The Diptera and non-insect relative abundance is calculated as the sum of the Diptera taxa and non-insect taxa divided by the total number of organisms in the sample. The relationship between a given biotic index and nutrient was explored via LOWESS, without predefined limits of acceptable ecological conditions (i.e., metrics of invertebrate community composition), and regression tree analysis was used to evaluate the environmental thresholds for TP or TN, where the biotic metric revealed the break point (the greatest degree of change)^56^. Such analysis was only applied to the taxonomic indices possessing significant relationships with nutrients (TP or TN)^16^. The basic data statistics were analyzed in Microsoft Excel 2010, and the multivariate statistical analyses were conducted in SPSS 19.0.

### Ethics statements

No specific permits were required for the described field studies; the sampling did not cause any disturbance to the environment or to the protected species at the sampling sites.
